# CrossLabFit: A novel framework for integrating qualitative and quantitative data across multiple labs for model calibration

**DOI:** 10.1371/journal.pcbi.1013704

**Published:** 2025-11-20

**Authors:** Rodolfo Blanco-Rodriguez, Tanya A. Miura, Esteban Hernandez-Vargas

**Affiliations:** 1 Department of Mathematics and Statistical Science, University of Idaho, Moscow, Idaho, United States of America; 2 Department of Biological Sciences, University of Idaho, Moscow, Idaho, United States of America; Universidade de Vigo, SPAIN

## Abstract

The integration of computational models with experimental data is a cornerstone for gaining insight into biomedical applications. However, parameter fitting procedures often require a vast availability and frequency of data that are challenging to obtain from a single source.

Here, we present a novel methodology called “CrossLabFit”, which is designed to integrate data from multiple laboratories, overcoming the constraints of single-lab data collection. Our approach harmonizes disparate qualitative assessments, ranging from different experimental labs to categorical observations, into a unified framework for parameter estimation. By using machine learning clustering, these qualitative constraints are represented as dynamic “feasible windows” that capture significant trends to which models must adhere. For numerical implementation, we developed a GPU-accelerated version of differential evolution to navigate the cost function that integrated quantitative and qualitative information.

We validate our approach across a series of case studies, demonstrating significant improvements in model accuracy and parameter identifiability. This work opens a new paradigm for collaborative science, enabling a methodological roadmap to combine and compare findings between studies to improve our understanding of biological systems and beyond.

## Author summary

Understanding complex biological processes often requires building mathematical models. These models can simulate how cells, tissues, or populations behave, but they must be calibrated to data. In practice, calibration typically uses measurements from a single experiment, the primary dataset that the model aims to explain. Adding additional information can improve parameter estimates, but due to biological variability, data collected across experiments or labs are rarely aligned point-by-point. Even so, they carry trustworthy cues within ranges, for example, that a signal stays elevated over a time window, shows a single peak, or remains within certain range of values.

CrossLabFit formalizes how to use these additional data by converting them into feasible windows that guide parameter estimation alongside the primary dataset, without requiring exact numerical agreement at every time point in the auxiliary data. This enables integration of heterogeneous datasets across labs while respecting known variability. By combining conventional measurements with feasible windows, CrossLabFit narrows the plausible parameter space and yields models that are more robust and faithful to biological behavior.

This approach broadens the use of existing experimental knowledge, accelerating research across domains from cellular signaling to infectious disease dynamics and ecology, especially when data is distributed across labs and formats.

## Introduction

Computational modeling is a fundamental endeavor that enables the exploration of elusive biological phenomena. Mathematical models have unknown parameters whose estimation is of paramount importance. Without accurate parameter values, the model’s predictive value is compromised [1]. Rigorous parameter estimation not only verifies the model’s efficacy, but also refines our understanding of the biological phenomena in question [2].

Maximum likelihood estimators find values of the model parameters that give the observed data the highest probability of occurring under the assumed statistical model [3]. This can be achieved by maximizing the profile likelihood or minimizing a cost function, such as the residual sum of squares, where the best parameter set lies at the minimum of the cost function landscape. A computational technique employing profile likelihood was introduced by Raue et al. [4] to determine model parameters in ordinary differential equations based on experimental data. This method also enables the detection of structural and practical non-identifiability [5].

A common practice in the modeling community is reducing model complexity to tackle identifiability problems, however, this dramatically limits the holistic understanding of the biological problem [6]. Another approach is to integrate new datasets into the parameter fitting procedure [2]. Nevertheless, acquiring new data is a daunting and expensive endeavor, often constrained by resource limitations and experimental feasibility. Another common and debatable practice in the modeling community is combining datasets from different research articles. Even when laboratories rely on available biological standards to allow for reproducible quantification [7], quantitative inconsistencies still arise.

Datasets in biology cannot be compared straightforwardly between laboratories in a quantitative form, and it is not necessarily because of the stochasticity of biology [8]. For example, cell quantification by flow cytometry data is dependent on cell collection and staining techniques [9], and quantification of specific mRNAs by quantitative PCR is dependent on reaction conditions. Data from these examples are also affected by instruments and user-defined settings [10].

Quantification of virus concentrations provides another example of the difficulty in deriving accurate quantitative values that can be directly compared across studies [11]. Knowing the concentration of virus stocks is essential for calculating accurate dosages for infection studies and measuring the effectiveness of antiviral compounds. Within studies, these values can be reported as relative concentrations compared to values from control samples, but they are rarely comparable between studies [12]. Variability between laboratories, and even between replicate assays within the same lab, can hinder direct comparison of plaque assay data. Such differences may arise from factors including cell line passage history, assay protocol details, cell density, and experimental error, all of which can influence plaque formation and affect the accuracy and consistency of plaque visualization and counting [13].

The use of qualitative knowledge, such as thresholds, peaking time, and monotonicity regions, emerges as a potential approach to increase the accessibility of experimental data. Mitra et al. [14] proposed a methodology that combined qualitative and quantitative data. Their methodology hinges on constructing a unified objective function. Within this framework, a conventional quantitative term, expressed as the standard sum of squares over all data points, coexists with a qualitative term. This qualitative component incorporates observations in the form of inequality constraints, penalizing the cost function accordingly. This approach was implemented in the pyBioNetFit toolbox [15] and extended with Bayesian inference [16]. Another way to integrate data is by using monotonic relationships between the experimental measure and simulated model data, a concept known as optimal scaling [17,18]. Optimal scaling was achieved by categorizing data and generating surrogate data while preserving the monotonicity observed in experimental data [19]. Integrating gradient-based methods with qualitative data significantly improves the accuracy and speed of parameter estimation [20,21]. This approach has been implemented in the Python Parameter EStimation TOolbox (pyPESTO) [22].

Despite these approaches, qualitative behavior is grossly defined to upper or lower bounds, *e.g.,* qualitative knowledge is converted into inequality constraints imposed on the outputs of the model [14] or to categories [20]. Additionally, it is assumed that this additional information comes from the same lab group’s experiments. However, there is a wealth of untapped datasets in the scientific literature that can be merged to define temporal qualitative categories. For example, data from influenza infection within the host is used to estimate mechanistic parameters of infection and immune responses, and to allow forecasting of future dynamics [23–26]. Nonetheless, interactions with different arms of the immune system would require additional data [27–30], which is very unlikely to all come from the same lab.

Here, we propose a new methodology based on machine learning to generate qualitative constraints by merging experimental data from different laboratories. Based on the generated qualitative constraints, we apply a novel approach called “CrossLabFit”. In this approach, the cost function is divided into one fitting quantitative data and the other penalizing deviations from the unobserved variables. This approach allows us to limit the distributions in model parameters and limit model trajectories from those that are not realistic.

## Results

**Fig 1:**
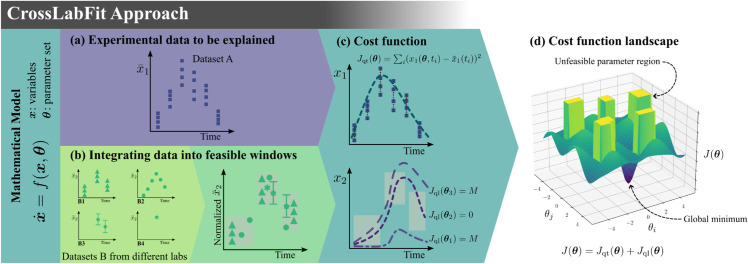
CrossLabFit approach. (a) We first consider an experimental dataset (dataset A) to be explained. (b) Additional information from various sources (datasets B) is collected to build feasible window constraints. (c) By incorporating these constraints into the cost function, (d) we transform the cost function landscape to enhance the search for the global minimum.

The main idea behind CrossLabFit is to model a dataset of interest (panel (a) of Fig 1) by minimizing a cost function, *e.g.*, residuals. As the data we want to explain is very likely limited to a few variables of the proposed model, we introduce an approach that integrates additional datasets from different labs and sources into dynamic domains where the model trajectories should reside (panel (b) of Fig 1). We refer to this dynamic domain as “feasible windows”. These feasible windows act as qualitative constraints that delineate the search parameter space by penalizing the cost function on model trajectories that do not pass through a window (panel (c) of Fig 1). Feasible windows will refine the cost function landscape for the global minimum while limiting model trajectories that have unrealistic biological behavior (panel (d) of Fig 1). By using different testbed models, we will show the efficacy of the integrative cost function next.

### Integrative cost function

While our CrossLabFit approach can be applied to any computational model, for presentation purposes, we will consider here a mathematical model expressed with Ordinary Differential Equations (ODEs) to study a biological problem in the following form

$$\dot{x}=f(x,\theta ).$$ (1)

Model variables x are defined by equations that depend on these variables and a set of parameters $\theta =(\theta_{1},\theta_{2},\ldots ,\theta_{n})$. We typically fit our model by finding the minimum of a cost function which measures the difference between our simulated variable *x*_*i*_ and the empirically observed data ${\hat{x}}_{i}$. This cost function corresponds to a landscape in a hyperspace ${\mathbb{R}}^{n}$, where *n* is the number of parameters in θ.

The essence of our approach lies in the optimization routine, which requires the reformulation of the cost function to include qualitative knowledge. The resulting cost function is a composite of quantitative and qualitative elements given by

$$J(\theta )=J_{\text{qt}}(\theta )+J_{\text{ql}}(\theta )$$ (2)

where $J_{\text{qt}}$ and $J_{\text{ql}}$ represent the cost functions corresponding to the quantitative and qualitative parts, respectively. For the quantitative part, we could consider the Residual Sum of Squares (RSS) in the following form

$$J_{\text{qt}}(\theta )=\text{RSS}(\theta )=\sum_{i,j}{\left({\hat{x}}_{j}(\theta ,t_{i})-x_{j}(t_{i})\right)}^{2}$$ (3)

where ${\hat{x}}_{j}$ is the observed variable of the model, *x*_*j*_ is the empirical data of the variable *j*, and *t* is the time.

We incorporate the qualitative component into the cost function by adding penalties when model trajectories fall outside predefined feasible windows. At this point, we define these feasible windows. For each variable *k* that is not part of the quantitative fit, we define *w* feasible windows as bounded regions in time and in the domain of the variable *k*. These are represented by a vector of feasible windows $W^{\;k}$, where each window is defined as:

$$W_{i}^{\;k}=[t_{i}^{\;\text{l}},t_{i}^{\;\text{u}}]\times [x_{i}^{\;\text{l}},x_{i}^{\;\text{u}}{]}^{\;k}$$ (4)

Here, $t_{i}^{\;\text{l}}$ and $t_{i}^{\;\text{u}}$ are the lower and upper time bounds, and $x_{i}^{\;\text{l}}$ and $x_{i}^{\;\text{u}}$ are the corresponding bounds in the domain of the variable *k*. These windows act as qualitative constraints, and the associated qualitative cost function is defined as:

$$J_{\text{ql}}(\theta )=\left\{ \begin{array}{cc} 0 & \;\text{if }{\hat{x}}^{\;k}(\theta ,t)\cap W_{i}^{\;k}\neq \emptyset \text{ for all }i=1,\ldots ,w\\ M & \;\text{otherwise, where }M\gg J_{\text{qt}}(\theta ) \end{array}\right.$$ (5)

where ${\hat{x}}^{\;k}$ represents the model-predicted trajectory of variable *k*, and *M* is a large penalty factor, chosen to be much greater than the quantitative cost $J_{\text{qt}}$. The constraint is satisfied as long as the trajectory intersects each feasible window at least once.

The goal of parameter estimation is to identify the parameter set that minimizes this integrative cost function, ideally reaching the global minimum. This can be expressed as:

$$\theta^{\;\text{best}}=\underset{\theta }{\text{arg min}}J(\theta )$$ (6)

Panel (d) of Fig 1 illustrates how the qualitative component of the cost function modifies the cost function landscape. The rectangular barriers represent the penalized space. These high barriers in the landscape eliminate model trajectories, preventing further exploration in those areas. In addition, these barriers not only speed up the search for the best parameter set, but also exclude parameter sets that do not correspond to realistic biological behavior, thus improving parameter identifiability.

It is worth noting that the quantitative term $J_{\text{qt}}$ is proportional to the negative log-likelihood under the assumption of independent Gaussian measurement errors, while the qualitative term $J_{\text{ql}}$ acts as a penalty enforcing feasibility windows. The resulting integrative cost function can therefore be interpreted as a penalized likelihood, where the penalty term incorporates prior qualitative knowledge. Since the combined function is not a pure likelihood in the classical sense, subsequent use of bootstrapping and profile likelihood methods should be viewed as operating on a pseudo-likelihood.

### Building feasible windows from data among different labs

The main question now is how to construct these feasible windows with experimental data from different laboratories. When we obtain data from different labs, we often encounter discrepancies in the quantitative values of the observables and the timing due to different measurement start points. However, we can rely on qualitative trends within specific ranges of values and time-frames.

Panel (b) of Fig 1 illustrates different examples of the types of data, *e.g.*, replicates, higher frequency measurements, confidence intervals, or even a single data point. Datasets can be mapped into a shared space where different datasets can be grouped into categories, which we named “feasible windows”. These windows can vary in size and shape and cover a range of time and values.

The primary challenge in building feasible window constraints is comparing and merging datasets from different labs as they may have different value scales. An approach is to normalize each dataset using its maximum and minimum values and combine all data into a qualitative space between 0 and 1. To compare our model predictions in this normalized space, we propose to rescale the feasible windows from the normalized space to the maximum value expected from our system. This approach preserves the shape of the observable data, regardless of their absolute values. As a result, data with slightly different scales but similar qualitative trends can be treated equivalently.

The second challenge is to determine the shape and size of the windows. Small windows lead to more constrained trajectories in the qualitative cost function, resulting in a more limited search parameter space for the optimizer algorithm. Conversely, large windows render the qualitative constraints ineffective by allowing the entire parameter search space. The shape of the windows also affects the barriers in the parameter search space.

We use rectangular windows for computational simplicity, as it is easier to determine whether simulation values fall within a specific range rather than within another geometry, *e.g.,* circles. The task is to determine the number of windows and how they should span the dataset. Our proposed approach involves four steps presented in Algorithm 1, and it is more objective than manually placing windows, although manual placement is also a potential option.

**Algorithm 1 Pipeline to build feasible window constraints.**



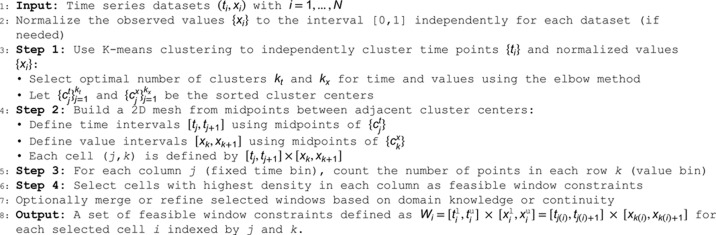



### Quantitative datasets A and auxiliary datasets B

To use our approach, we require two types of datasets. The first type is used to quantitatively fit the model using the quantitative cost function defined in Eq (3). We refer to this dataset as dataset A. In principle, multiple datasets (e.g., A1, A2, ...) can be used in this role to fit different model variables quantitatively. However, in many biological applications, only one such dataset is commonly available.

The second type of data, used to define feasible window constraints as described in Algorithm 1, is referred to as datasets B (e.g., B1, B2, ...). These datasets are typically obtained from independent experiments or external sources and are not directly paired with dataset A. While dataset A provides the values to be matched in the model fitting, datasets B serve to guide the qualitative behavior of additional model variables for which no direct measurements are available in dataset A. Importantly, the values in datasets B are not meant to be quantitatively matched by the model and therefore do not contribute to the quantitative part of the cost function (e.g., RSS). Instead, they inform the construction of feasible window constraints that steer the optimization toward biologically plausible solutions. Using multiple datasets for a given variable helps better capture the qualitative trends and dynamic features of the system.

### Benchmark to evaluate performance

In this section, we present the results of the cycle Lotka-Volterra model to illustrate the potential of the CrossLabFit approach. Supplementary Material presents more complex models to show the applicability of our method. The cycle Lotka-Volterra model is represented by the following equations


$$\dot{X_{1}}=-a_{1}X_{1}-a_{2}X_{1}X_{2}+a_{3}X_{1}X_{3}$$

$$\dot{X_{2}}=a_{4}X_{1}X_{2}-a_{5}X_{2}-a_{6}X_{2}X_{3}$$

$$\dot{X_{3}}=-a_{7}X_{1}X_{3}+a_{8}X_{2}X_{3}-a_{9}X_{3}$$ (7)

where *X*_*i*_ is a variable state, for each state *i*, and each *a*_*i*_ is a parameter of the model; the other two variations are presented in S1 and S2 Figs. The model represents a cyclic interaction among three species, with the third species influencing the first, creating a feedback loop. This model serves as a testbed to assess the efficacy of the CrossLabFit approach when qualitative constraints are synergized with quantitative data.

We generated the two types of datasets for this nine-parameter Lotka-Volterra model: dataset A and auxiliary datasets B. First, we assigned ground truth values to all parameters and solved the model equations to obtain the reference (ground truth) dynamics. Dataset A was then constructed by sampling variable *X*_1_ at regular intervals and adding log-normal noise to each data point.

**Fig 2:**
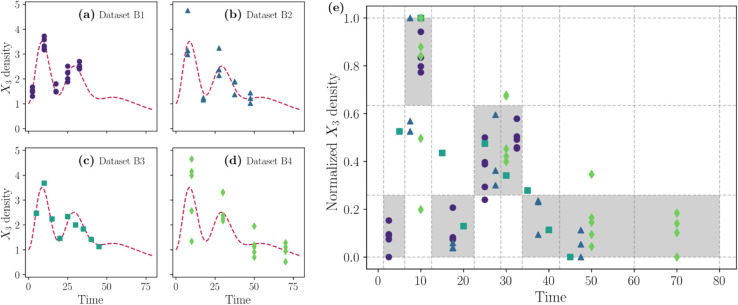
Building feasible window constraints. Panels (a)-(d) show the four synthetic datasets (B1-B4) with different frequencies, replicates, and deviation. Panel (e) shows the datasets merged in a normalized space, where the grid shows the number of vertical cells determined by clustering the *X*_3_ value and the number of horizontal cells is determined by clustering the time dataset. The feasible windows are chosen according to the maximum number of points between cells in the same column.

For datasets B, we created four synthetic datasets (B1–B4) for *X*_3_ using the ground truth solution, each with different sampling frequencies, numbers of replicates, and levels of variation between replicates. These datasets were then processed using Algorithm 1 to generate the feasible window constraints, as illustrated in Fig 2.

Using the synthetic datasets and feasible window constraints, we estimated the nine parameters with a custom Differential Evolution (DE) algorithm. In a previous study [1], we compared multiple optimization algorithms under identical conditions and found comparable performance. DE was selected here for its robustness to local minima, ease of implementation, and suitability for parallelization, making it a practical and efficient choice for our framework. Other optimizers produced similar results (S3 Fig), indicating that our approach is largely independent of the optimization method. However, because the cost function includes hard penalties, a non–gradient-based optimizer is required. While a soft penalty could be used, it would introduce additional hyperparameters, whereas our current formulation performs well without this added complexity.

**Fig 3:**
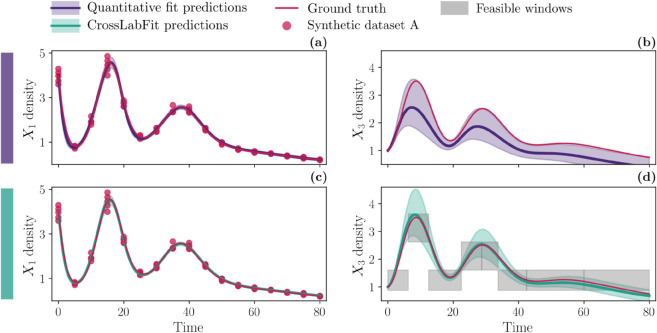
Dynamics of cycle Lotka-Volterra model for the two different approaches. A comprehensive comparison of model predictions for two approaches: the standard quantitative approach (purple) and using our CrossLabFit approach (turquoise). In the panel of plots, each column represents the variables *X*_1_ and *X*_3_. The first row shows the results of the standard approach results using synthetic data with log-normal noise, highlighted by red circles. The last row integrates feasible window constraints for *X*_3_. The ground truth is denoted by a solid red line, contrasted with a color-coded solid line and shaded area showing the median and confidence interval, respectively, of the simulations obtained by estimating parameters using nonparametric bootstrapping, for each approach.

Fig 3 shows the plot panel with the model results. Each column represents the model variables *X*_1_ and *X*_3_, while each row corresponds to different strategies. The top row illustrates parameter fitting that involves quantitative parameter estimation using only *X*_1_ synthetic data (represented by red circles). This is a common scenario in which only one dataset is available to fit a single model variable; we refer to this as the standard quantitative approach, which serves as the basis for comparison with our method. In the second row, feasible window constraints are integrated for the variable *X*_3_ during the optimization process. The solid red lines represent the ground truth solutions, while the solid purple and turquoise lines show the median simulations. The shaded areas indicate the confidence intervals obtained from the best parameter estimates using our DE optimizer combined with nonparametric bootstrapping.

S4 Fig contains plots for the variable *X*_2_ and strategies related to adding feasible windows constraints for *X*_2_. We also used the same strategies with the other two Lotka-Volterra models in S1 and S2 Figs.

Numerical results demonstrate the impact of incorporating qualitative constraints into the parameter estimation process. Parameter fitting that relies solely on quantitative data serves as the baseline. Adding qualitative constraints to *X*_3_ significantly improves the fit for this variable, as shown by the tight shaded area around the solid red ground truth line in panel (d) of Fig 3. Adding qualitative constraints only on *X*_2_ does not markedly improve predictions for *X*_2_ or *X*_3_ (S4 Fig). This is likely because *X*_2_ already interacts with the fitted variable *X*_1_ through a subtractive, lower-amplitude coupling (*a*_2_), allowing the optimizer to adjust *X*_3_ indirectly via *a*_1_ and *a*_3_. In contrast, *X*_3_ couples additively to *X*_1_ with larger amplitude, so constraints on *X*_3_ more directly restrict parameter combinations. Since *X*_1_ data already constrain parameters affecting *X*_2_, adding *X*_2_ constraints offers little extra benefit, explaining why constraining both *X*_2_ and *X*_3_ yields results similar to constraining *X*_3_ alone (S4 Fig).

In general, these results suggest that the stochastic DE optimizer is more effective when qualitative constraints are integrated into the optimization process, leading to a more accurate representation of the underlying dynamics. The inclusion of qualitative constraints likely narrows the feasible parameter space, thereby accelerating the convergence of the optimization algorithm to the true parameter values.

**Fig 4:**
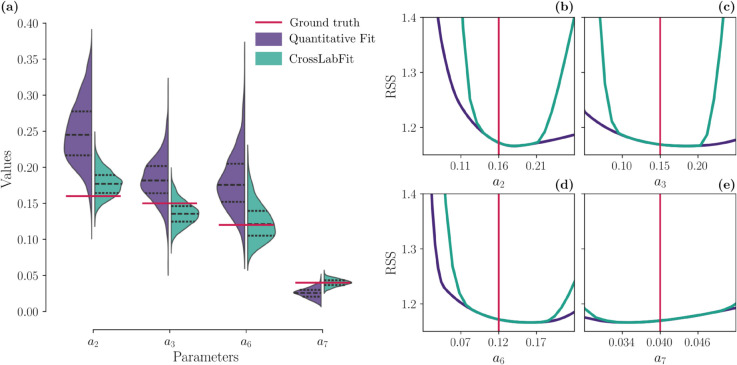
Parameter distribution and likelihood profiles for cycle Lotka-Volterra model. Panel (a) shows violin plots illustrating the variability and density of the estimated parameters *a*_2_, *a*_3_, *a*_6_, and *a*_7_ derived from 1000 bootstrap resamples across the two data integration strategies. The width of each violin indicates the sample density at different values, with a red line marking the ground truth. Dashed lines within each violin represent the median and interquartile range. Panels (b)-(e) show likelihood profiles for four parameters in the Lotka-Volterra model (*a*_2_, *a*_3_, *a*_6_, and *a*_7_), comparing two data integration strategies (standard and CrossLabFit approach) against the ground truth to assess parameter identifiability. These panels plot RSS against parameter values, indicating that minima closer to the ground truth represent more accurate estimates.

In panel (a) of Fig 4, we show violin plots illustrating the variability and distribution of the estimated parameter obtained by 1000 bootstrapping resamples using the two different data integration strategies. The width of each violin represents the density of bootstrap samples at different values, providing a visual comparison of the variability of the estimated parameter across the two strategies. The red line shows the ground truth values for each parameter. In each violin plot, the median and interquartile interval are marked as dashed lines.

A tighter concentration of bootstrap samples around the ground truth line indicates a more accurate and consistent estimation process. The variations in the width and centering of the violins for each strategy illustrate how the inclusion of qualitative constraints for *X*_3_ affects the parameter estimates. The integration of qualitative constraints brings the estimates for the parameters *a*_2_, *a*_3_, *a*_6_ and *a*_7_ closer to the ground truth, with reduced variance, indicating improved estimation accuracy. In particular, the estimates for the *a*_6_ and *a*_7_ parameters are visibly improved, with the median being aligned with the ground truth values. In contrast, as observed in S4 Fig, the quantitative estimation for *a*_8_ is sufficient, as the median is close to the ground truth. For the remaining parameters, the addition of qualitative constraints does not show any significant improvement, except for a reduction in the variance of the distributions.

Panels (b)-(e) of Fig 4 show the likelihood profiles for four parameters (*a*_2_, *a*_3_, *a*_6_ and *a*_7_) of the testbed model. Each panel shows the RSS as a function of parameter values, with the two different strategies superimposed to illustrate their effect on parameter identifiability. The vertical red lines mark the ground truth values, which serve as a reference for the accuracy of each strategy. To obtain these curves, we chose a parameter and set its value at regular intervals throughout the search space; for each parameter value, we estimated the rest of the parameters using our DE optimizer and obtained the minimum RSS. The closer the minimum of the curves is to the ground truth, the more robust the parameter estimation is with the given strategy.

Overall, the likelihood profiles incorporating qualitative constraints are tighter than those using the standard quantitative approach, resulting in narrower confidence intervals. The profile for *a*_3_ becomes more accurate with the inclusion of *X*_3_-related qualitative data, and is closer to the ground truth. This improvement is expected given the role of *a*_3_ in the interaction between *X*_1_ and *X*_3_. The estimation of the parameters *a*_2_ and *a*_6_ also benefits from the qualitative constraints, showing bounds closer to the true values, while the quantitative estimation yields only a left bound. For *a*_7_, our approach shows a discernible minimum. For the remaining parameters shown in S4 Fig, there is no apparent improvement, with some still showing structural non-identifiability problems. Nevertheless, the closer bounds to the ground truth suggest that our approach still offers advantages.

### Case study: CD8+ T cell response to influenza infection

**Fig 5:**
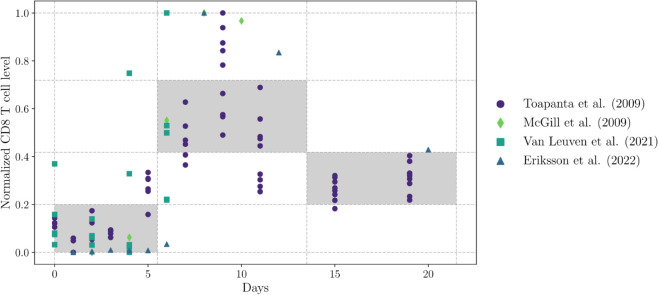
Building feasible window constraints for CD8+ T cell dynamic. The data were normalized using the minimum and maximum values of each dataset. The plot shows the data from the four sources and the feasible window constraints formed by a grid, where the number of vertical cells is determined by clustering the CD8+ T cell data and the number of horizontal cells is determined by clustering the days dataset. The windows were chosen according to the maximum number of points between cells in the same column.

To exemplify this approach with real data, we consider immunological responses to influenza infection (Fig 5). We generated feasible window constraints using CD8+ T cell data from four different sources [27–30]. We collected data from studies using consistent methods to measure CD8+ T cells in the lungs of BALB/c mice intranasally infected with influenza A PR8(H1N1). The doses administered varied between studies, and each dataset covered different frequencies and ranges of days.

Fig 5 shows the four datasets and the feasible window constraints generated using Algorithm 1. Briefly, each dataset was normalized to its own minimum and maximum values. Clustering was then performed independently on the temporal axis and the normalized value axis. For each axis, we tested different numbers of clusters and selected the optimal count for splitting the data. Using the midpoints between cluster centers, we constructed a mesh of cells. For each column in this mesh, the cell with the highest data point density was selected as a feasible window, ensuring better representation of the CD8+ T cell dynamics.

We applied our approach to a mechanistic model of influenza A infection [31,32]. For the quantitative part of the cost function, we used the viral dataset from mouse lung tissues reported in [33], which serves as the quantitative data the model seeks to reproduce. The equations are written as follows:

$$\dot{U}=-\beta UV$$ (8)

$$\dot{I}=\beta UV-\delta_{I}TI$$ (9)

$$\dot{V}=pI-cV$$ (10)

$$\dot{T}=s_{T}+rTV-\delta_{T}T$$ (11)

*U* represents the population of uninfected target cells, *I* denotes the population of infected cells, *V* is the concentration of virus particles, and *T* represents the population of CD8+ T cells. The parameters include the infection rate *β*, which describes how effectively the virus infects uninfected cells; the rate $\delta_{I}$ at which T cells kill infected cells; the replication rate *p* of new virus particles by infected cells; the clearance rate *c* of free virus particles; the proliferation rate *r* of T cells in response to the presence of the virus; and the natural death rate $\delta_{T}$ of T cells. The homeostatic proliferation of CD8+ T cells is $s_{T}=\delta_{T}T(0)$, maintaining a positive level of these cells even after the virus is cleared. Here, *T*(0) represents the initial condition of T cells, while the initial infected population is set to zero.

We performed parameter estimation using two approaches: the standard quantitative approach [34] that relies solely on the viral dataset, and our new CrossLabFit approach, which combines the viral dataset with feasible window constraints from Fig 5. For the CrossLabFit approach, we assumed a maximum T cell level of 10^7^, scaling and shifting the feasible windows to fit within the range of 10^6^ to 10^7^. Additionally, we imposed two constraints: first, a maximum allowable T cell level to prevent simulations from exceeding the upper limit, and second, a minimum viral load level to prevent viral dynamics from rebounding once the minimum is reached. The minimum detectable viral load is approximately 100 TCID50 (50% tissue culture infectious dose) [35]; we used 50 TCID50 as both the initial viral load and the minimum threshold for viral load constraint.

This mathematical model includes six parameters and four initial conditions. Given the high degree of freedom relative to the amount of data available for quantitative fitting, we fixed commonly reported parameters and the initial conditions using values from the literature. Consequently, four parameters, *β*, $\delta_{I}$, *p*, and *c*, were estimated, as they are directly related to the viral dynamics. The fixed values and the search bounds can be found in Table 3 of the Materials and Methods section.

**Fig 6:**
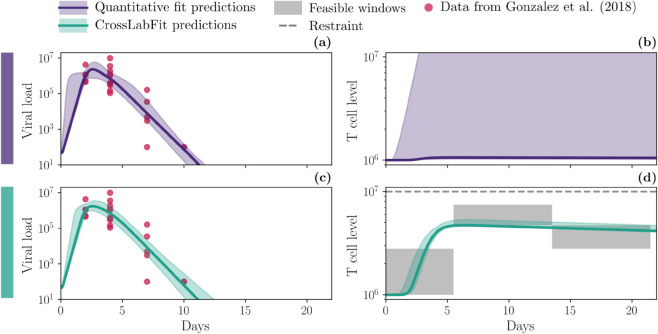
Dynamics of influenza model for the two different approaches. A comprehensive comparison of influenza model predictions for two approaches: the standard quantitative approach (purple) and using our CrossLabFit approach (turquoise). In the panel of plots, each column shows the viral dynamics *V* (panels (a) and (c)) and T cell dynamic *T* (panels (b) and (d)). The first row shows standard approach results using viral load data, highlighted by red circles. The last row integrates feasible window constraints for *T*. Color-coded solid line and shaded area illustrate the median and confidence interval, respectively, of the simulations obtained by estimating parameters using nonparametric bootstrapping, for each approach.

Fig 6 compares the influenza model predictions from the two approaches. In the panel of plots, each column shows the viral dynamics *V* (panels (a) and (c)) and CD8+ T cell dynamics *T* (panels (b) and (d)). The first row presents the results of the standard approach using viral load data, indicated by red circles. The second row integrates feasible window constraints for *T* and threshold constraints for both *V* and *T*, shown as gray dashed lines. The solid lines represent the median, and the shaded areas indicate the confidence intervals of the simulations, which were obtained from parameter estimates using non-parametric bootstrapping.

Both approaches exhibit similar behavior for viral dynamics, with comparable confidence intervals. However, the CD8+ T cell dynamics differ significantly. The standard approach shows a wide confidence interval, with the median CD8+ T cell level remaining close to the initial value. In contrast, the CrossLabFit approach produces a well-defined curve for CD8+ T cell dynamics with a much narrower confidence interval.

**Fig 7:**
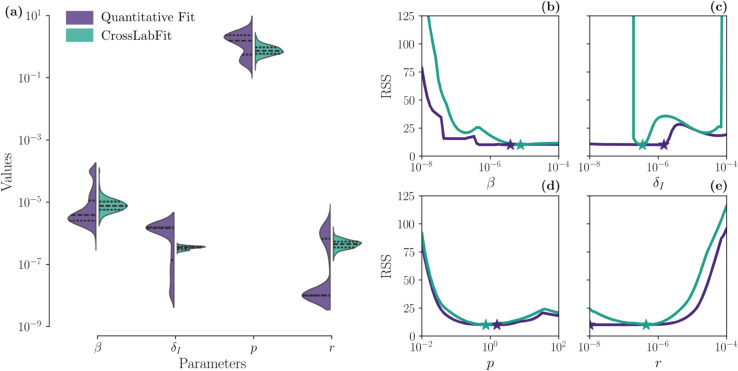
Parameter distribution and likelihood profiles for influenza model. Panel (a) shows violin plots illustrating the variability and density of the estimated parameters *β*, $\delta_{I}$, *p*, and *r* derived from 1000 bootstrap resamples across the two data integration strategies. The width of each violin indicates the sample density at different values. Dashed lines within each violin represent the median and interquartile range. Panels (b)-(e) show likelihood profiles for four parameters in the influenza model (*β*, $\delta_{I}$, *p*, and *r*), comparing two data integration strategies ( the standard and our CrossLabFit approaches) to assess parameter identifiability. The stars indicate the median values from the parameter distributions obtained via bootstrapping, with colors corresponding to the respective approach.

Panel (a) of Fig 7 presents violin plots illustrating the variability and density of the estimated parameters *β*, $\delta_{I}$, *p*, and *r* derived from 1000 bootstrap resamples across the two data integration strategies. The width of each violin reflects the sample density at different values, while the dashed lines represent the median and interquartile range. Notably, the CrossLabFit approach shows a tighter distribution of values, indicating improved confidence intervals, particularly for $\delta_{I}$. Additionally, the parameter *r* exhibits a bimodal distribution under the standard quantitative estimation, in contrast to the unimodal distribution observed with the CrossLabFit approach.

Panels (b)-(e) of Fig 7 present likelihood profiles for four parameters in the influenza model (*β*, $\delta_{I}$, *p*, and *r*), comparing the standard approach with the CrossLabFit approach to assess parameter identifiability. The stars indicate the median values from the parameter distributions obtained via bootstrapping, with colors corresponding to their respective approach. For parameters *β* and *p*, there is no significant improvement in resolving the practical non-identifiability issue. While *β* shows a slightly improved left bound, *p* remains similar for both approaches. In contrast, parameters $\delta_{I}$ and *r* show notable improvements with the CrossLabFit approach. The profile likelihood for $\delta_{I}$ shifts from structural non-identifiability to having a clear minimum with tighter bounds, although an additional minimum is still observed. The parameter *r* shows a slight improvement in the left bound compared to the standard approach. These results are expected, as $\delta_{I}$ and *r* are directly related to CD8+ T cell dynamics in the model equations. Therefore, incorporating additional information in the form of feasible windows from an unobserved variable helps identify parameters associated with that observable.

Table 1 presents the estimated parameters for the influenza A model in mouse lungs, comparing the standard approach with the CrossLabFit approach. The values shown are the medians and 95% confidence intervals from the bootstrapped estimates displayed in panel (a) of Fig 7. In addition to differences in the median values, we observe a significant improvement in the confidence intervals with the CrossLabFit approach. Notably, parameter *β* lacks an upper confidence bound in the standard approach, as the upper value reaches the limit of the search bounds. Similarly, for parameter $\delta_{I}$, the lower confidence bound is restricted by the lower search limit. For parameter *r*, the median lies at the lower bound of both the confidence interval and the search range in the standard approach. In contrast, the CrossLabFit approach provides well-defined confidence intervals for all parameters, also a narrower range for parameter *p* compared to the standard approach. Thus, the CrossLabFit approach enhances the ability to define confidence intervals for all estimated parameters.

**Table 1:** Comparison of estimated parameters for the influenza A model in mouse lungs. Values are median and 95% confidence interval from bootstrapped estimates.

Parameter	Standard approach	CrossLabFit approach
*β* ($\times 10^{-6}$)	3.87 [1.45, 100]	7.92 [3.47, 23.7]
$\delta_{I}$ ($\times 10^{-6}$)	1.47 [0.01, 1.71]	0.35 [0.25, 0.41]
*p*	1.57 [0.23, 4.20]	0.73 [0.39, 1.51]
*r* ($\times 10^{-8}$)	1 [1, 178]	44.2 [23.1, 78.3]

## Discussion

The integration of qualitative knowledge into the quantitative parameter estimation process has yielded promising results in our study. Incorporating additional information as qualitative constraints improves the search for a minimum in the cost function by excluding certain regions of the parameter space. Numerical results revealed that the CrossLabFit approach achieved a closer fit to the ground truth compared to the standard approach. As shown in Fig 3, the dynamic of variable *X*_3_ was successfully replicated with the CrossLabFit approach. This suggests that incorporating qualitative constraints can effectively narrow the parameter search space, leading to more accurate model predictions and improving identifiability analysis. Such findings align with previous research that emphasizes the value of qualitative information in complex system modeling [14,20,21].

Our approach aims to capture the qualitative trends in the dynamics of different datasets. While the values in these datasets may not be directly compatible with the experimental data to be explained, certain features, such as the timing of peaks, are expected and can be used to constrain the variable space to plausible dynamics. This is conceptually similar to the work of Mitra et al. [14], but in our case, the qualitative information is derived from additional quantitative datasets. Besides, unlike the approach of Oguz et al. [36], which applies penalties directly in the parameter space, our method imposes penalties on the time evolution of model variables. Consequently, the feasible region in parameter space is not explicitly defined and may be highly irregular. In principle, their parameter-space penalty approach could be combined with ours, but this would require sufficient prior knowledge about the model parameters.

Bootstrapping analysis in panel (a) of Fig 4 revealed that the variability of parameter estimates generally decreased when qualitative constraints were included. However, the accuracy of the estimated values is improved only for the parameters *a*_6_ and *a*_7_, since the medians hit the ground truth value. This enhancement in the precision of parameter estimates is particularly notable for parameters that directly interact with the variable *X*_3_ for which feasible window constraints were generated. Likelihood profiles in panels (b)-(e) of Fig 4 also indicated that some parameters associated with qualitative constraints had tighter likelihood profiles, demonstrating the improvement of identifiability.

For the Lotka–Volterra model, we generated synthetic dataset A for quantitative fitting using a moderate noise level (small standard deviation) to represent a favorable scenario. This allowed us to assess parameter estimation performance using both the standard quantitative approach and our method. To further evaluate robustness, we tested increasing noise levels in dataset A. As shown in S5 Fig, higher noise reduced prediction accuracy, as expected. Nevertheless, across all noise levels, incorporating window constraints consistently improved the prediction of variable *X*_3_.

Our approach has some limitations. It relies on a hard-penalty method, where violations of feasible windows result in binary penalties. This requires confidence in window placement and may exclude valid parameter sets, reducing robustness. The discontinuities in the cost function also necessitate the use of non–gradient-based optimizers. While smoother penalty functions (e.g., logistic) could allow gradient-based optimization, our tests showed similar improvements for *X*_3_ but slower convergence and reduced accuracy for *X*_1_ (S6 Fig). Soft penalties also demand extra hyperparameter tuning and computational effort, and they make it more complex to implement our specific condition that a trajectory need only intersect the window at some point in time. For these reasons, we selected hard penalties with DE, which offered good performance with minimal tuning. Furthermore, the binary penalty function also works well with other non–gradient-based optimizers from the SciPy library without altering the cost function (S3 Fig), indicating that our approach can be readily implemented on different platforms.

Finally, our method does not improve identifiability for all parameters. For example, *a*_5_, associated with the decay rate of *X*_2_, shows little improvement, likely due to structural non-identifiability. In general, adding qualitative constraints to *X*_2_ does not enhance parameter estimation. Nevertheless, the improvements observed for other parameters and in dynamic predictions justify the use of our approach.

S1 and S2 Figs include results for two additional Lotka-Volterra testbed models. For the linear-chain Lotka-Volterra model without a feedback loop, parameter estimation enhancements were predominantly observed for the qualitative constraints on *X*_3_. Conversely, in the modified Lotka-Volterra model, where *X*_2_ and *X*_3_ interact solely with *X*_1_ and not with each other, the integration of qualitative constraints for both *X*_2_ and *X*_3_ yielded improvements in parameter estimation.

In our study, the algorithm to build feasible windows plays a crucial role in integrating diverse data sources into the parameter estimation process. We chose to normalize each dataset independently, as the varying scales between datasets posed a challenge. Specifically, larger datasets tended to overshadow smaller ones, making it difficult to reflect the dynamics of the smaller datasets in the model. By normalizing each dataset to its minimum and maximum values, we ensured that all data sources were given equal weight in the feasible window construction. In Fig 5, we can observe all the normalized data using this approach. When we attempted to normalize the datasets as a group, however, we found that the data from references [27,28] had larger values, which flattened the dynamics of the other datasets.

We acknowledge that alternative strategies could also be considered. For example, normalizing relative to baseline values at time zero would be appropriate if all datasets shared a reliable baseline, while log-transforming could mitigate scale differences and emphasize relative changes, provided all values are positive. Both approaches, however, would alter the interpretation of the feasibility window bounds. Another consideration is that datasets with more samples per time point may disproportionately influence window definition, as denser sampling contributes more points to the clustering process. While this can reflect the greater information content of such datasets, it may also create imbalance when sampling density is driven by experimental design rather than biological variability. Weighting datasets or points inversely by their sampling density could help address this, and is a potential refinement for future work. Although these alternatives were not studied here, they remain promising directions, particularly when datasets are more directly comparable.

We also applied our approach to influenza infection dynamics in the lungs of mice (Fig 6). While the improvement in predicted dynamics may appear modest (CD8+ T cell dynamics is nearly flat in the standard approach), the main advantage lies in the gains in parameter identifiability and confidence. Although not all parameters benefit, the improvement for some is substantial. For example, in Fig 7, the results for $\delta_{I}$ show a clear advantage: panel (a) demonstrates a much narrower parameter distribution with our method, and panel (c) reveals a well-defined likelihood minimum absent in the standard approach. This increases confidence in the uniqueness of the $\delta_{I}$ estimate and strengthens the interpretation of its role in the clearance of infected cells. This study is intended as a proof of concept using a well-established influenza infection model. While more complex models could also benefit, assessing improvements in identifiability becomes more challenging as the number of parameters increases, and such analyses is beyond the scope of this study.

This study is a proof of concept using Lotka–Volterra models as testbeds and an influenza infection model as a practical application. To explore applicability to more complex systems, we also applied the framework to a glycolysis model with oscillatory dynamics [37] (S1 File). Fitting one variable quantitatively and imposing feasible windows on three others, we observed modest but consistent improvements in dynamics (Fig A in S1 File) and tighter bootstrap parameter distributions, including a median matching the ground truth for one parameter (Fig B in S1 File). Although the gains may appear modest, these improvements enhance confidence in parameter estimates and, consequently, strengthen mechanistic inference.

One might assume that incorporating additional datasets in a standard quantitative approach would yield results comparable to using feasible window constraints. However, directly fitting multiple datasets often amplifies inconsistencies. To illustrate this, the middle row in S7 Fig shows a scenario where the Lotka-Volterra model was simultaneously fit to both *X*_1_ and *X*_3_ using synthetic data from datasets A and B1–B4. Due to noise and mismatched temporal frequencies, the optimizer favored parameter sets that overfit high-frequency fluctuations, resulting in suboptimal dynamics. Similarly, we directly fit both the viral load and the T cell data to the influenza model (S8 Fig). This resulted in nearly flat T cell dynamics with reduced variation compared to fitting the viral load alone. These examples highlight the risk of directly incorporating discordant datasets and the advantage of our window-based method, which selectively filters out noise while preserving key qualitative trends, thereby guiding the optimization process in a more robust and interpretable way.

Our findings underscore the selective utility of integrating data from different labs into qualitative constraints for constructing more complex models. Numerical results on testbed models highlight that the key to improving parameter estimation lies not only in the quantity of information, but also in the time position of the qualitative constraint and the strategic selection of variables for qualitative integration. This pioneering approach will lead to significant improvements in computational biology.

## Materials and methods

### Testbed model and synthetic datasets.

The model shown in Fig 3 is one of three Lotka-Volterra models, each incorporating different interactions between the observable variables *X*_1_, *X*_2_, and *X*_3_. The parameters *a*_*i*_ for i∈{0,…,9} define the different models. In the model shown in Fig 3, the input term $a_{0}X_{1}$ was omitted from the rate of change equation for ${\dot{X}}_{1}$ because its value was set to zero. For each model, we selected arbitrary parameter values designed to produce damped oscillations with peaks and valleys; a complete list of parameters is provided in Table 2.

**Table 2:** Parameters for the three Lotka-Volterra models used to generate synthetic data.

Parameter	Linear	Cycle	2-predators
*a*_0_	0.14	0.00	0.45
*a*_1_	0.00	0.18	0.00
*a*_2_	0.16	0.16	0.16
*a*_3_	0.00	0.15	-0.15
*a*_4_	0.15	0.11	0.15
*a*_5_	0.11	0.02	0.11
*a*_6_	0.05	0.12	0.00
*a*_7_	0.00	0.04	-0.08
*a*_8_	0.06	0.12	0.00
*a*_9_	0.05	0.02	0.05

Synthetic dataset A for *X*_1_ were generated using these parameter values by solving the ODEs with the odeint function from the SciPy library. We sampled the resulting dynamics of each observable every 5 time units. For each sampled point, we generated 5 random samples using a log-normal distribution. This approach was chosen for two reasons: first, it introduces greater variation at higher values, which is typical of real biological data; and second, it avoids generating negative values, which would not make sense in this system. The datasets B were generated in a similar manner, but with variations in sampling frequency, number of samples, and the standard deviation of the log-normal noise distribution. All data generation, analysis, and plotting was done in Python, while parameter estimation and bootstrapping was done for our custom DE optimizer coded in CUDA/C.

### Materials for modeling influenza infection.

The viral dataset used for quantitative model fitting was obtained from our own lab, as reported in Gonzalez et al. [33] (Fig 2A, Mock/PR8). To construct the feasible window constraints, we used raw datasets from Toapanta et al. [27] (Fig 9B, Adult mice) and from our own data in Van Leuven et al. [30] (Fig 6J, Mock/PR8). Two additional datasets were extracted using the PlotDigitizer web app [38] from McGill et al. [28] (Fig S1A) and Eriksson et al. [29] (Fig S3B).

Each dataset used to build the feasible windows, was independently normalized using its own minimum and maximum values before being combined into a single dataset. We then applied Algorithm 1 using the KMeans clustering function from the Scikit-learn library with default parameters. Clustering was performed separately on the time and normalized value axes, with the number of clusters varied to determine the optimal count via the elbow method. The resulting window boundaries were saved to a file and provided as input to our custom-built DE optimizer.

The mathematical model contained six parameters, two of which were fixed to reference values reported in the literature. The remaining four parameters were estimated using the two approaches presented in this work, with our custom DE implementation in CUDA/C, designed to exploit parallelization and accelerate computations for bootstrapping and likelihood profile generation. Table 3 lists the reference values and search bounds used in our DE optimizer for each model parameter, and the initial conditions for each model variable.

**Table 3:** Parameter values for the influenza model.

Parameter	Fixed Value	Search Bounds
*β*	-	[10^−8^,10^−4^]
$\delta_{I}$	-	[10^−8^,10^−4^]
*p*	-	[10^−2^,10^2^]
*c*	2.9 (ref [39])	-
*r*	-	[10^−8^,10^−4^]
$\delta_{T}$	0.011 (ref [32])	-
*U*(0)	10^7^ (ref [31])	-
*I*(0)	0	-
*V*(0)	50	-
*T*(0)	10^6^ (ref [31])	-

### High performance computing.

The code for bootstrapping and identifiability analysis was implemented in CUDA/C to leverage high-performance computing on the Falcon supercomputer [40], utilizing GPU nodes. Falcon is located at the Idaho National Laboratory Collaborative Computing Center (C3) in Idaho Falls, Idaho, USA. We performed 1000 optimizations for each case study and each approach, and 50 runs for each likelihood profile. Each optimization required approximately 1–5 minutes to complete.

### GPU-accelerated version of differential evolution optimizer.

To optimize the cost function, we employed a custom-built Differential Evolution (DE) algorithm [41], chosen for its simplicity and effectiveness in various applications [42]. We developed a GPU-accelerated version of the DE algorithm using CUDA/C, significantly enhancing computational speed by leveraging GPU parallelization, following the approaches of previous studies [43,44].

Additionally, we implemented a fifth-order Runge-Kutta method to solve the ODE system within our optimizer. Within the DE algorithm, we utilized a population array consisting of 8192 parameter sets and 10000 iterations for the main loop. The initial population was randomly sampled from a uniform distribution spanning the lower and upper bounds of each parameter. To generate a new mutant vector within the population, we adopted the DE/rand/1/bin strategy. This approach involves selecting three random vectors (sets of parameters) from the population and using them to mutate each vector within the population. The mutation was performed with a mutation factor of *F*_*m*_ = 0.8, a crossover rate of *C*_*r*_ = 0.8, and a binomial selection criterion. A detailed description of the algorithm is provided in Algorithm A in S2 file.

In our GitHub repository, we provide two implementations of the CrossLabFit framework. The CUDA/C version, which was used for the parameter estimation results reported in this study, is optimized for high performance and parallelization on GPU hardware. In addition, we include a Python version based on SciPy optimizer, which is more user-friendly and can be readily used to run tests or adapt the approach to new models on standard desktop or laptop computers.

### Bootstraping.

We applied a nonparametric bootstrap approach using Monte Carlo resampling [45]. Data were resampled with replacement to create a sample of the same size as the original dataset. Parameters were then estimated from each resampled dataset. We ran 1000 bootstrap parameter estimates for each strategy. This allowed us to obtain the corresponding parameter distributions by refitting the model in each of these iterations.

### Identifiability analysis.

A model is considered identifiable when its parameters can be uniquely determined. We used the profile likelihood method [4], where each parameter is varied while the others are re-optimized. This approach detects both structural and practical non-identifiability [4,46]. Structural non-identifiability arises from the model structure, while practical non-identifiability is due to data quality or quantity. A parameter is identifiable if its profile likelihood is concave; a flat valley indicates practical non-identifiability, and a constant cost function suggests structural non-identifiability.

## Supporting information

S1 Fig Results for the linear Lotka-Volterra model. The panel displays a sketch of the model, the equations used, simulation results for each strategy, parameter distributions, and likelihood profiles comparing the strategies. The strategies involve parameter estimation using synthetic data from *X*_1_ alone, with qualitative constraints in *X*_2_, in *X*_3_, and in both *X*_2_ and *X*_3_. All plots are color-coded according to the key labels above for consistency across strategies.
(TIFF)

S2 Fig Results for the 2-predator Lotka-Volterra model. The panel displays a sketch of the model, the equations used, simulation results for each strategy, parameter distributions, and likelihood profiles comparing the strategies. The strategies involve parameter estimation using synthetic data from *X*_1_ alone, with qualitative constraints in *X*_2_, in *X*_3_, and in both *X*_2_ and *X*_3_. All plots are color-coded according to the key labels above for consistency across strategies.
(TIFF)

S3 Fig Dynamics of the Lotka-Volterra model using different non-gradient-based optimizers. Simulation results compare two parameter estimation strategies: the standard approach (without window constraints) and the CrossLabFit approach (with window constraints). Each row corresponds to results obtained with a different optimizer from the SciPy library.
(TIFF)

S4 Fig Results for the cyclic Lotka-Volterra model. The panel displays a sketch of the model, the equations used, simulation results for each strategy, parameter distributions, and likelihood profiles comparing the strategies. The strategies involve parameter estimation using synthetic data from *X*_1_ alone, with qualitative constraints in *X*_2_, in *X*_3_, and in both *X*_2_ and *X*_3_. All plots are color-coded according to the key labels above for consistency across strategies.
(TIFF)

S5 Fig Dynamics of the Lotka-Volterra model using different levels of noise. Simulation results compare two parameter estimation strategies: the standard approach (without window constraints) and the CrossLabFit approach (with window constraints). Each row corresponds to results obtained with a different level of noise in the dataset A (small, medium, and high standard deviation).
(TIFF)

S6 Fig Dynamics of the Lotka-Volterra model using a smooth penalty function. Simulation results compare two parameter estimation strategies: the standard approach (without window constraints) and the CrossLabFit approach (with window constraints). Feasible window constraints were implemented using a logistic penalty function, and optimization was performed with a gradient-based method.
(TIFF)

S7 Fig Dynamics of the cyclic Lotka-Volterra model. The panel presents simulation results for the three state variables, with each column representing a different variable. The rows compare three parameter estimation strategies: using synthetic data from *X*_1_ alone, incorporating raw data from *X*_3_, and applying the CrossLabFit approach.
(TIFF)

S8 Fig Dynamics of the influenza model. The panel presents simulation results for the influenza model, with each row comparing three parameter estimation strategies: using synthetic data from viral load alone, incorporating raw data from T cells, and applying the CrossLabFit approach.
(TIFF)

S1 File Glycolysis model results. The glycolysis model and the results of comparing two parameter estimation strategies are presented: the standard approach (without window constraints) and CrossLabFit (with window constraints).
(PDF)

S2 File Pseudocode for a GPU-accelerated Differential Evolution algorithm. The algorithm shows the pseudocode for a GPU-accelerated DE algorithm with feasible window constraints for parameter estimation.
(PDF)
