# Supplementary figures and images for: CrossLabFit: A novel framework for integrating qualitative and quantitative data across multiple labs for model calibration
Source: PLoS Comput Biol. 2025 Nov 20;21(11):e1013704. doi: 10.1371/journal.pcbi.1013704 (PMC12677793; doi:10.1371/journal.pcbi.1013704)

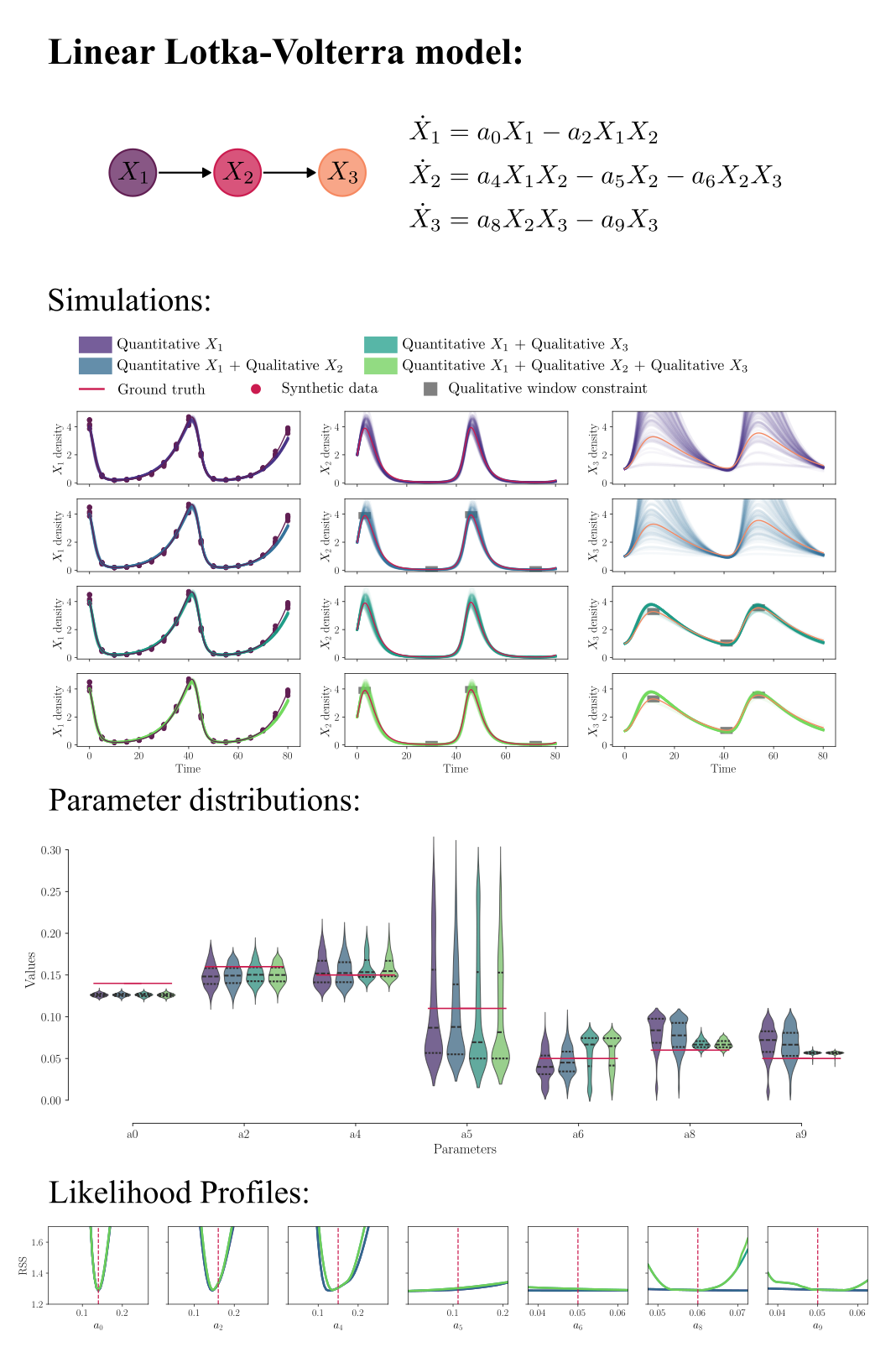

Supplement: S1 Fig — The panel displays a sketch of the model, the equations used, simulation results for each strategy, parameter distributions, and likelihood profiles comparing the strategies. The strategies involve parameter estimation using synthetic data from X1 alone, with qualitative constraints in X2, in X3, and in both X2 and X3. All plots are color-coded according to the key labels above for consistency across strategies. (TIFF) [file pcbi.1013704.s001.tif]

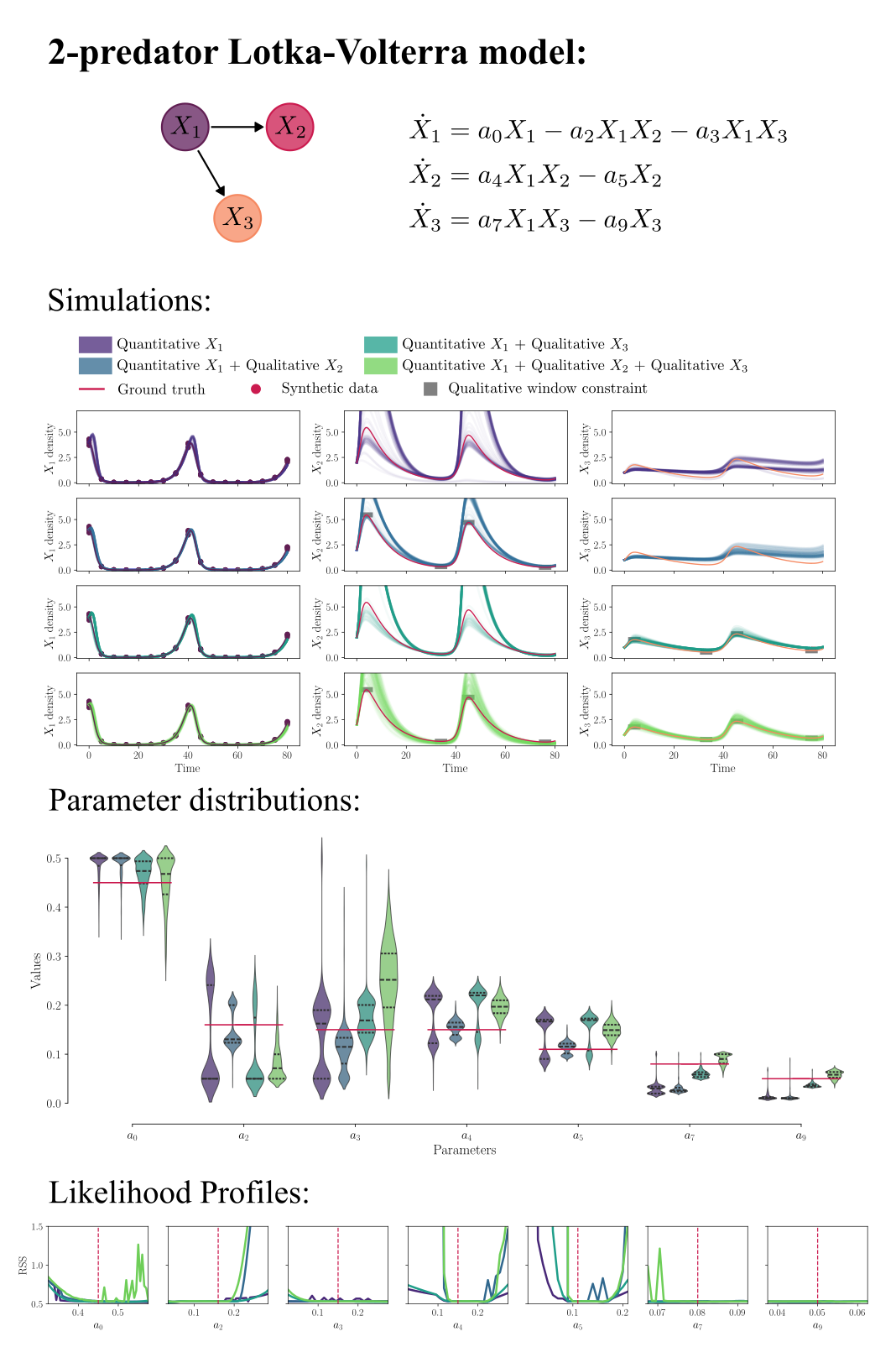

Supplement: S2 Fig — The panel displays a sketch of the model, the equations used, simulation results for each strategy, parameter distributions, and likelihood profiles comparing the strategies. The strategies involve parameter estimation using synthetic data from X1 alone, with qualitative constraints in X2, in X3, and in both X2 and X3. All plots are color-coded according to the key labels above for consistency across strategies. (TIFF) [file pcbi.1013704.s002.tif]

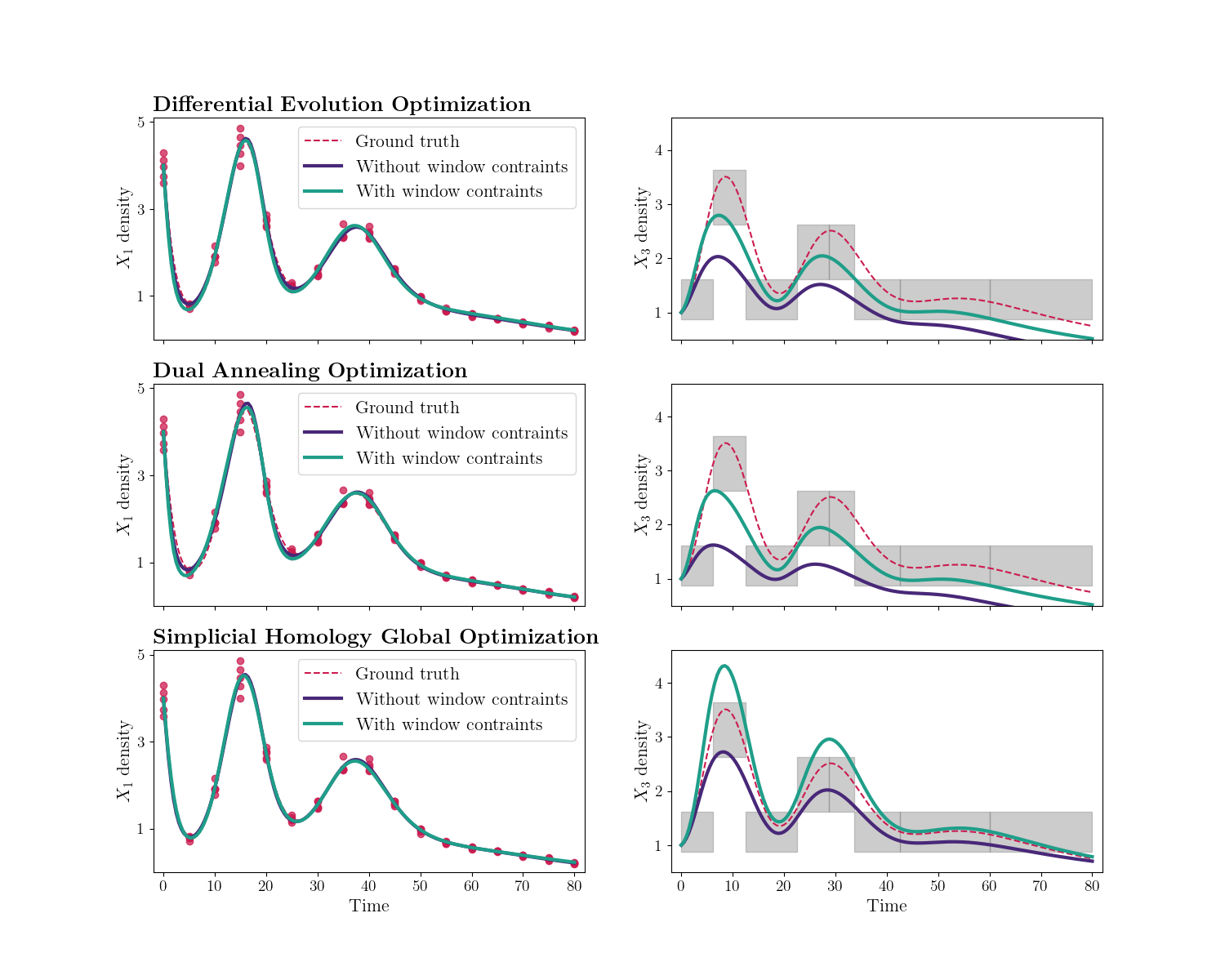

Supplement: S3 Fig — Simulation results compare two parameter estimation strategies: the standard approach (without window constraints) and the CrossLabFit approach (with window constraints). Each row corresponds to results obtained with a different optimizer from the SciPy library. (TIFF) [file pcbi.1013704.s003.tif]

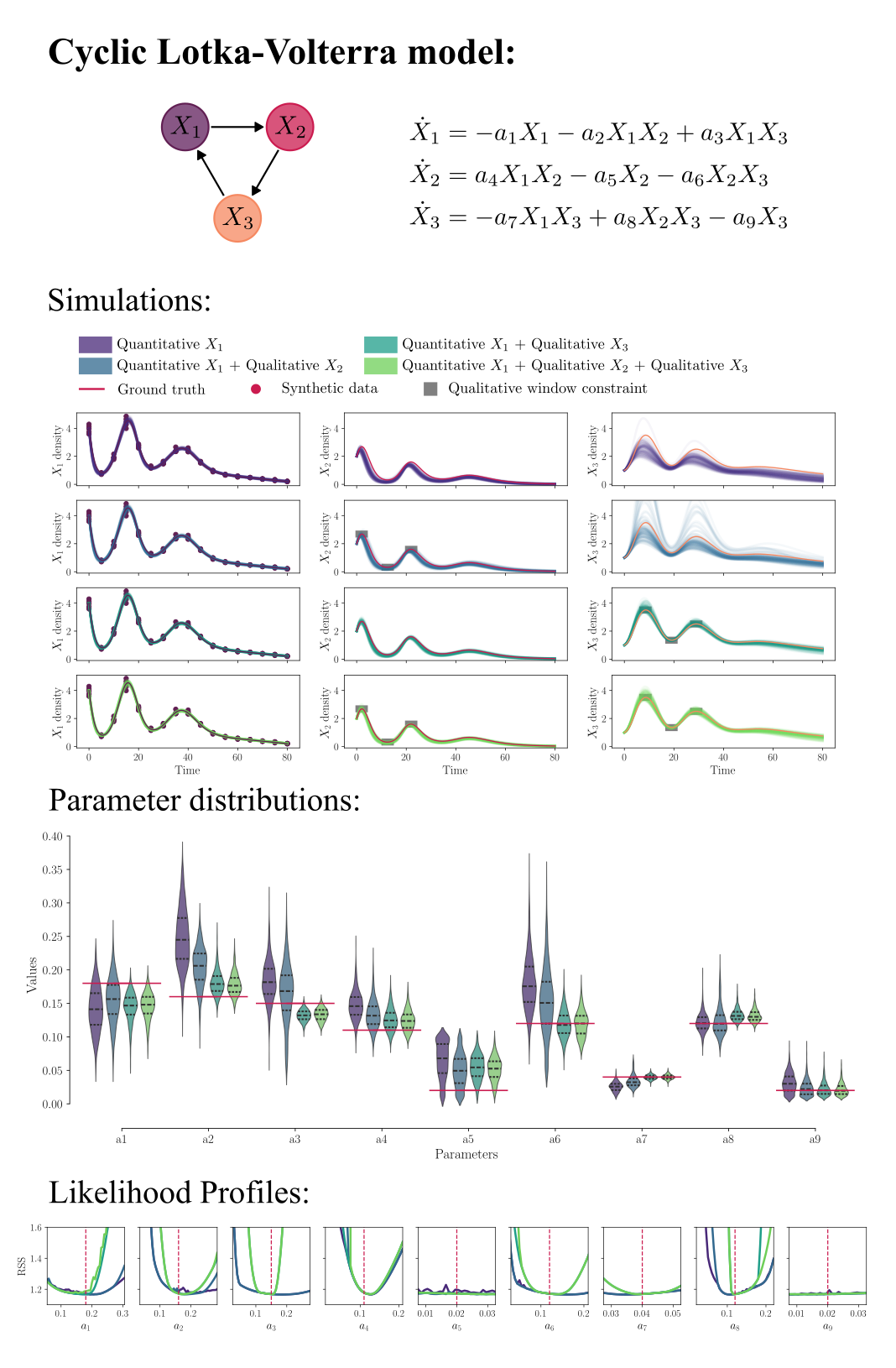

Supplement: S4 Fig — The panel displays a sketch of the model, the equations used, simulation results for each strategy, parameter distributions, and likelihood profiles comparing the strategies. The strategies involve parameter estimation using synthetic data from X1 alone, with qualitative constraints in X2, in X3, and in both X2 and X3. All plots are color-coded according to the key labels above for consistency across strategies. (TIFF) [file pcbi.1013704.s004.tif]

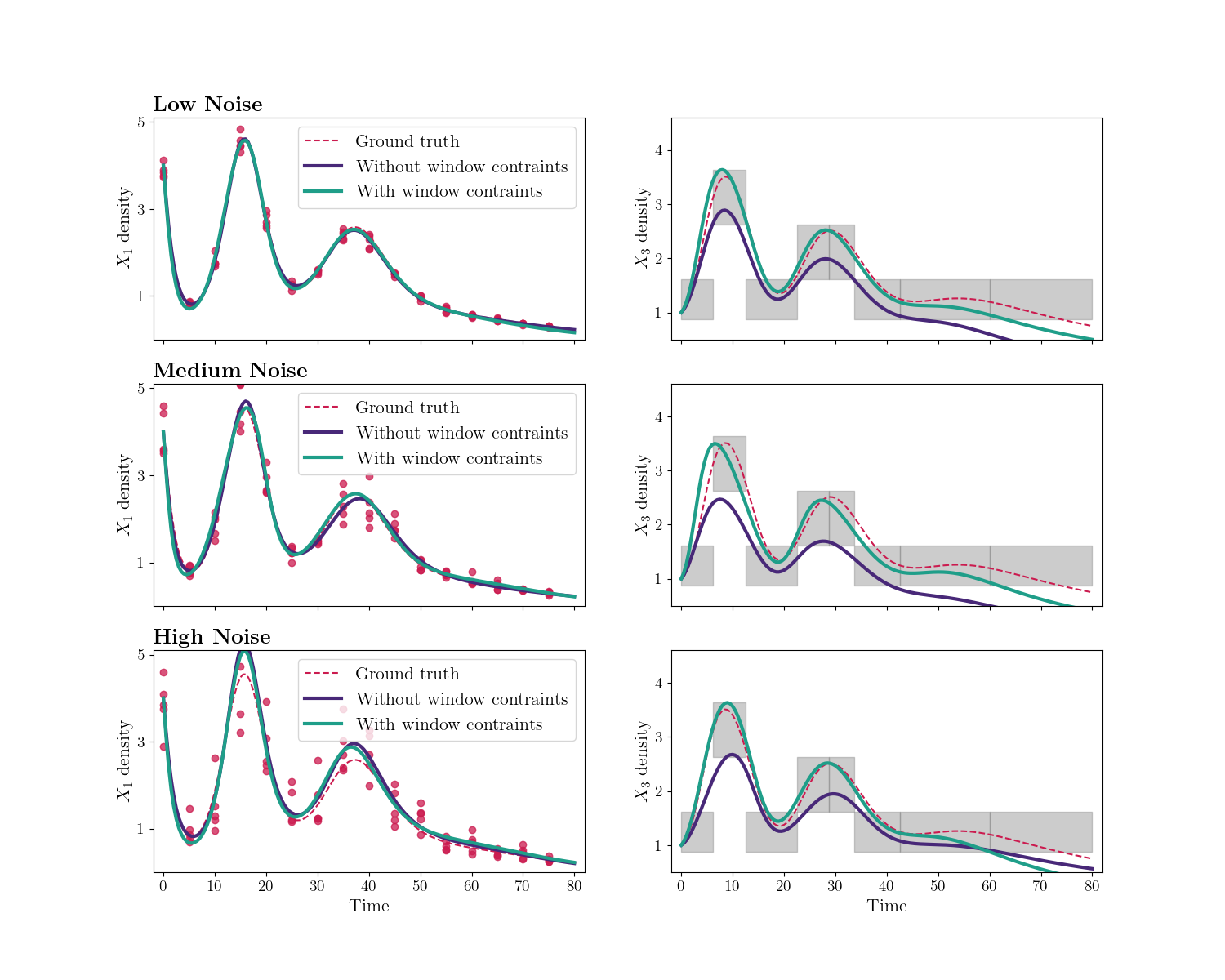

Supplement: S5 Fig — Simulation results compare two parameter estimation strategies: the standard approach (without window constraints) and the CrossLabFit approach (with window constraints). Each row corresponds to results obtained with a different level of noise in the dataset A (small, medium, and high standard deviation). (TIFF) [file pcbi.1013704.s005.tif]

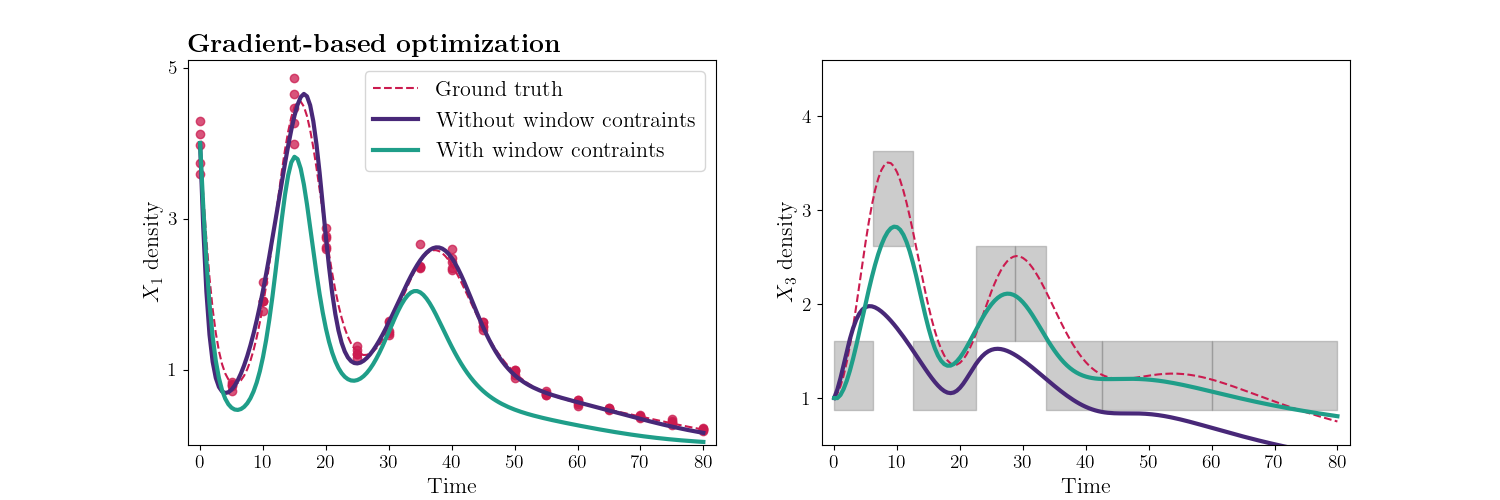

Supplement: S6 Fig — Simulation results compare two parameter estimation strategies: the standard approach (without window constraints) and the CrossLabFit approach (with window constraints). Feasible window constraints were implemented using a logistic penalty function, and optimization was performed with a gradient-based method. (TIFF) [file pcbi.1013704.s006.tif]

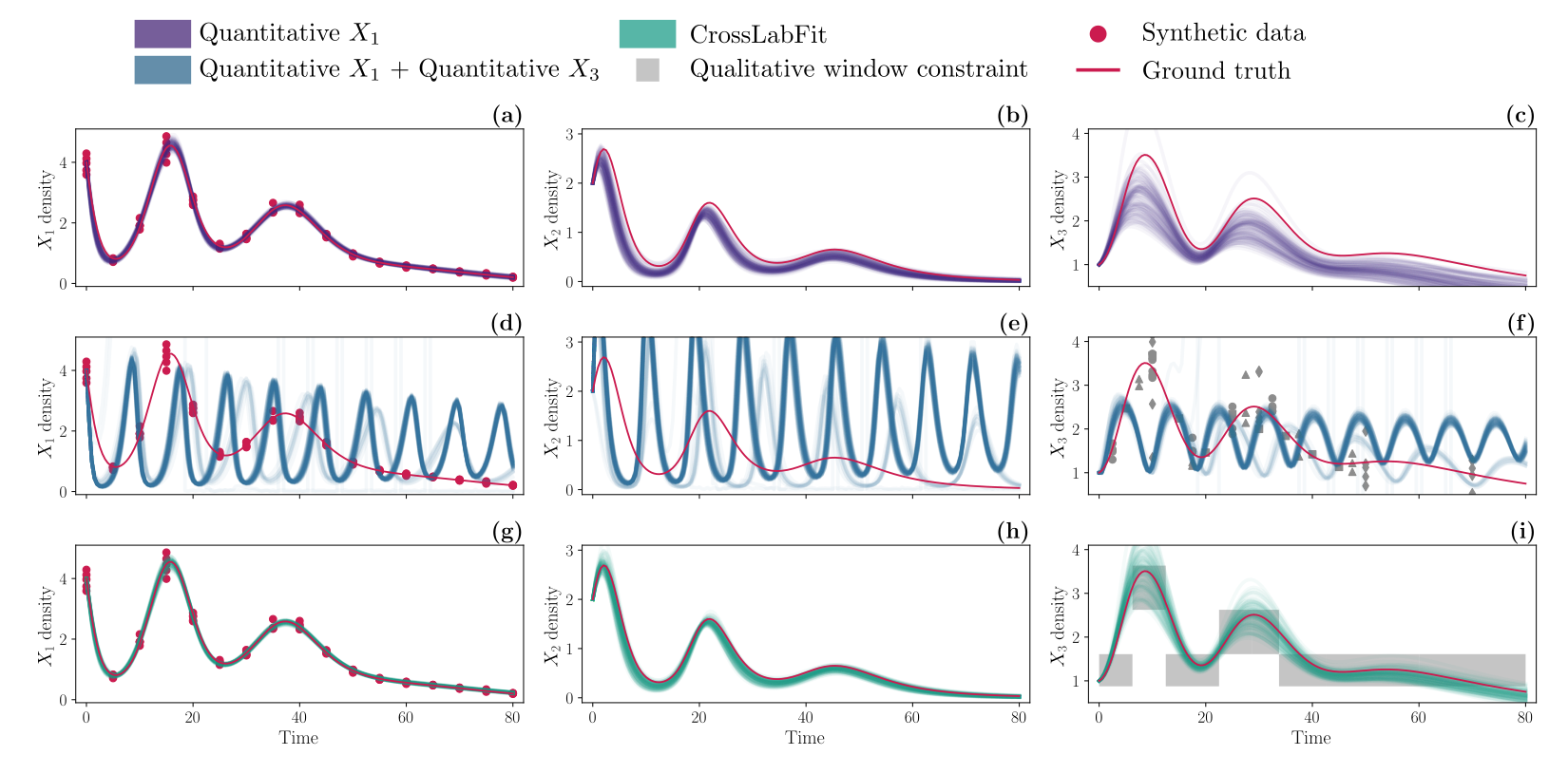

Supplement: S7 Fig — The panel presents simulation results for the three state variables, with each column representing a different variable. The rows compare three parameter estimation strategies: using synthetic data from X1 alone, incorporating raw data from X3, and applying the CrossLabFit approach. (TIFF) [file pcbi.1013704.s007.tif]

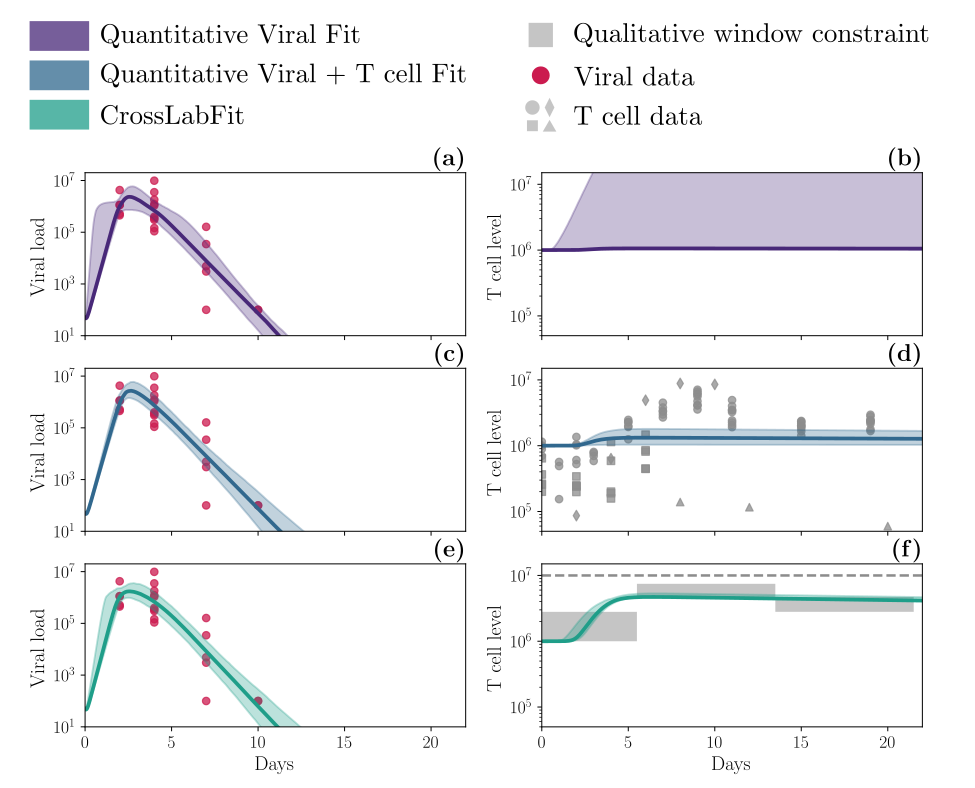

Supplement: S8 Fig — The panel presents simulation results for the influenza model, with each row comparing three parameter estimation strategies: using synthetic data from viral load alone, incorporating raw data from T cells, and applying the CrossLabFit approach. (TIFF) [file pcbi.1013704.s008.tif]
