# Supplementary material for: CrossLabFit: A novel framework for integrating qualitative and quantitative data across multiple labs for model calibration
Source: PLoS Comput Biol. 2025 Nov 20;21(11):e1013704. doi: 10.1371/journal.pcbi.1013704 (PMC12677793; doi:10.1371/journal.pcbi.1013704)
Supplement: S1 File — The glycolysis model and the results of comparing two parameter estimation strategies are presented: the standard approach (without window constraints) and CrossLabFit (with window constraints). (PDF) [file pcbi.1013704.s009.pdf]

# Glycolysis testbed model

The glycolysis model we use follows the formulation analyzed by Ruoff et al. [*] for temperature compensation in yeast glycolytic oscillations, with several reactions lumped into metabolite pools. $S_1$ denotes intracellular glucose; its dynamics $\dot{S}_1 = J_0 - v_1$ balance membrane influx $J_0$ and consumption by the lumped early glycolytic flux $v_1$. $S_2$ represents the triose-phosphate pool, $S_3$ 1,3-bisphosphoglycerate, and $S_4$ the pyruvate/acetaldehyde pool; $S_5$ is NADH, $S_6$ is ATP, and $S_7$ an extracellular coupling species. The core nonlinearity is the allosteric regulation of PFK captured in

$$v_1 = \frac{k_1 S_1 S_6}{1 + (S_6/K_1)^4},$$

i.e., cooperative inhibition of PFK by ATP (Hill coefficient 4). Total cofactor pools (NAD+, NADH) and (ADP+, ATP) couple via $(N, S_5)$ and $(A, S_6)$, respectively. Exchange between intra- and extracellular pools is modeled through terms proportional to $\kappa$ (scaled by $\psi$). Here, $K_1$ is the ATP inhibition constant, $\kappa$ reflects membrane permeability, and $k_{1,\ldots,6}$ are effective rate constants for the lumped reaction steps. The complete ODE model is given by:

$$\begin{aligned}
\dot{S}_1 &= J_0 - v_1, \\
\dot{S}_2 &= 2\,v_1 - k_2 S_2 (N - S_5) - k_6 S_2 S_5, \\
\dot{S}_3 &= k_2 S_2 (N - S_5) - k_3 S_3 (A - S_6), \\
\dot{S}_4 &= k_3 S_3 (A - S_6) - k_4 S_4 S_5 - \kappa (S_4 - S_7), \\
\dot{S}_5 &= k_2 S_2 (N - S_5) - k_4 S_4 S_5 - k_6 S_2 S_5, \\
\dot{S}_6 &= -2\,v_1 + 2 k_3 S_3 (A - S_6) - k_5 S_6, \\
\dot{S}_7 &= \psi\,\kappa (S_4 - S_7) - k\,S_7.
\end{aligned}$$

This ODE system captures the key regulatory feature of glycolytic oscillations, ATP's cooperative inhibition of PFK, together with coupling to energy cofactors and transmembrane exchange. This structure preserves the oscillatory behavior of glycolysis and provides a challenging test case for our framework due to its nonlinearity and oscillatory dynamics.

Ref [*]: Ruoff, P., Christensen, M. K., Wolf, J., & Heinrich, R. (2003). Temperature dependency and temperature compensation in a model of yeast glycolytic oscillations. Biophysical Chemistry, 106(2), 179-192.

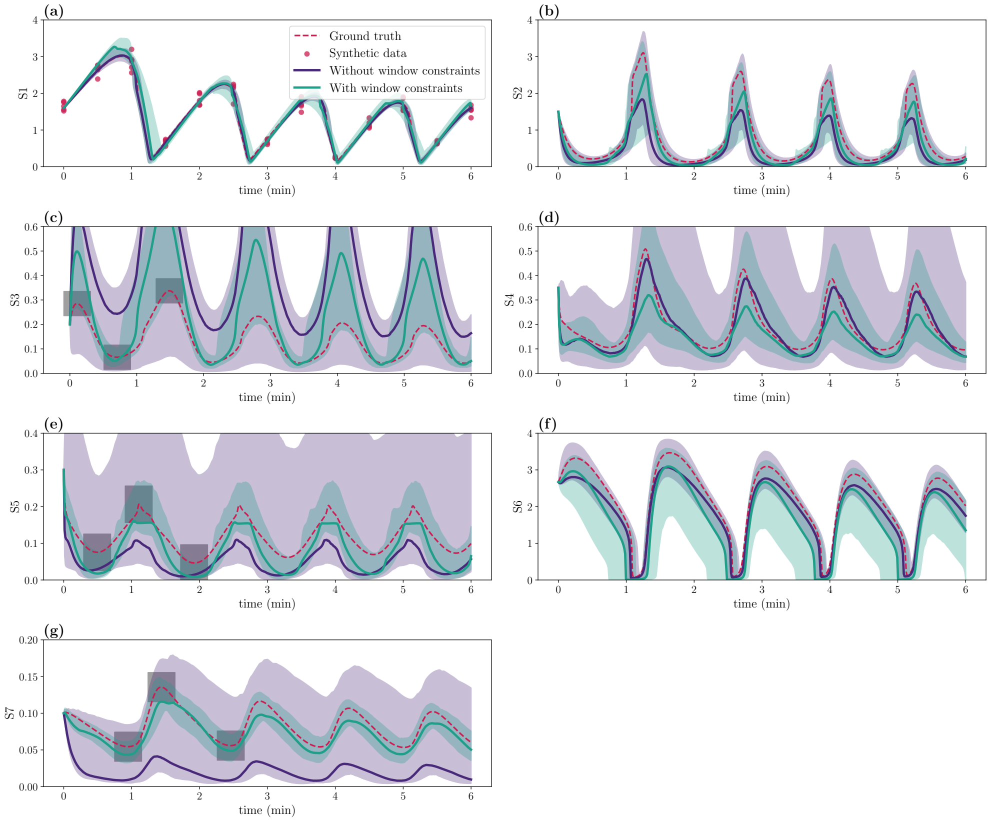


**Fig A.** Dynamics of the glycolysis model. Comparison of two parameter-estimation strategies: the standard approach (no window constraints) and CrossLabFit (with window constraints). Each panel shows the trajectory of one model variable under both strategies. Solid lines denote median simulations; shaded bands indicate bootstrap confidence intervals.

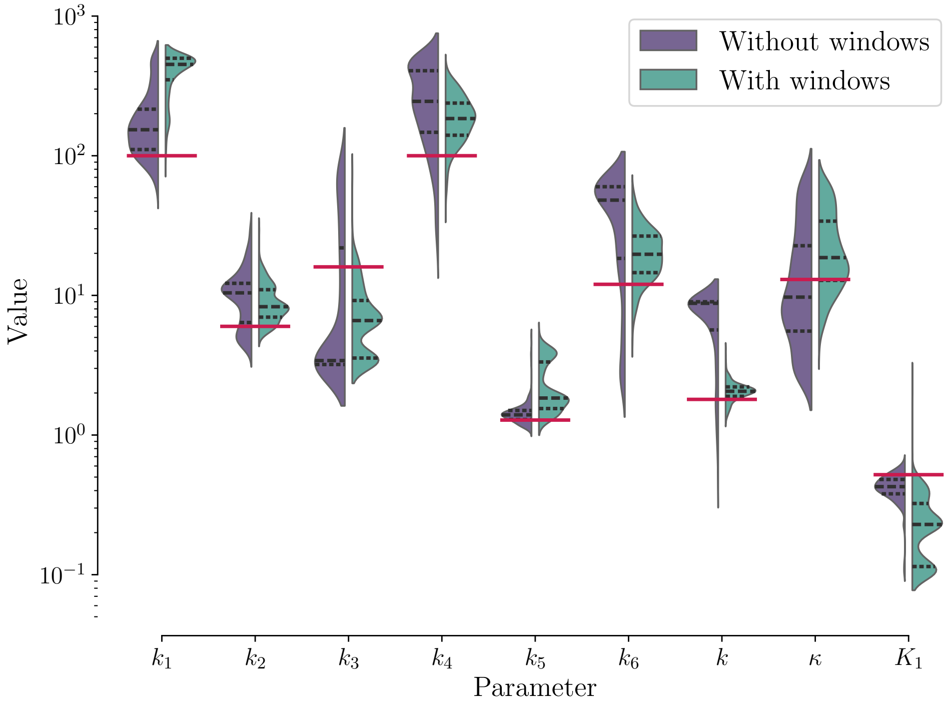


**Fig B.** Parameter distribution for glycolysis model. Figure shows violin plots illustrating the variability and density of the estimated parameters derived from 500 bootstrap resamples across the two data integration strategies. The width of each violin indicates the sample density at different values. Dashed lines within each violin represent the median and interquartile range.

**Table A.** Parameter values for the glycolysis model. The true values were taken from the reference [*] above.

| Parameter | True Value [*] | Search Bounds |
|---|---|---|
| $J_0$ | 2.5 | Fixed |
| $k_1$ | 100.0 | $[20, 500]$ |
| $k_2$ | 6.0 | $[1.2, 30]$ |
| $k_3$ | 16.0 | $[3.2, 80]$ |
| $k_4$ | 100.0 | $[20, 500]$ |
| $k_5$ | 1.28 | $[0.256, 6.4]$ |
| $k_6$ | 12.0 | $[2.4, 60]$ |
| $k$ | 1.8 | $[0.36, 9.0]$ |
| $\kappa$ | 13.0 | $[2.6, 65]$ |
| $K_1$ | 0.52 | $[0.104, 2.6]$ |
| $\psi$ | 0.1 | Fixed |
| $N$ | 1.0 | Fixed |
| $A$ | 4.0 | Fixed |
| $S_1(0)$ | 1.6 | Fixed |
| $S_2(0)$ | 1.5 | Fixed |
| $S_3(0)$ | 0.2 | Fixed |
| $S_4(0)$ | 0.35 | Fixed |
| $S_5(0)$ | 0.3 | Fixed |
| $S_6(0)$ | 2.67 | Fixed |
| $S_7(0)$ | 0.1 | Fixed |
