# Supplementary material for: CrossLabFit: A novel framework for integrating qualitative and quantitative data across multiple labs for model calibration
Source: PLoS Comput Biol. 2025 Nov 20;21(11):e1013704. doi: 10.1371/journal.pcbi.1013704 (PMC12677793; doi:10.1371/journal.pcbi.1013704)
Supplement: S2 File — The algorithm shows the pseudocode for a GPU-accelerated DE algorithm with feasible window constraints for parameter estimation. (PDF) [file pcbi.1013704.s010.pdf]

Algorithm A Pseudocode for a GPU-accelerated Differential Evolution algorithm with qualitative constraints for parameter estimation.

Input: N: population size, C_r: crossover probability, F_m: mutation factor, D: Number of parameters to estimate, M: penalty factor.

function COSTFUNCTION(Pars[i], Data_{qt}, Δt_{ql}, ΔX_{ql})
  Data_{sim} ← 5THRUNGE-KUTTASOLVER(ODEmodel, Pars[i])
  if Data_{sim} touches all qualitative windows defined for each square $\Delta t_{\text{ql}} \times \Delta X_{\text{ql}}$ **then**
    return $\sum_n (\text{Data}_{\text{sim}}[n] - \text{Data}_{\text{qt}}[n])^2$
  else
    return M
  end if
end function

Initialization: Generate a uniform random population vector Pars consisting of N arrays, each containing D parameters to estimate. Then, evaluate the cost function using the quantitative data Data_{qt} and qualitative boundary constraints, defined by Δt_{ql} (window time width) and ΔX_{ql} (window variable value height).
parfor $i \leftarrow 0$ to N **do**
  for $j \leftarrow 0$ to D **do**
    Pars[$iD + j$] ← uniform random value within the j search space
  end for
  J[i] ← COSTFUNCTION(Pars[i], Data_{qt}, Δt_{ql}, ΔX_{ql})
end parfor

Optimization: Main loop for optimization using the DE method with the mutation strategy DE/rand/1/bin.
for iterations ← 0 to maximum iterations **do**
  parfor $i \leftarrow 0$ to N **do** ▷ Each thread i of the GPU perform the code below
    $j_{\text{rand}} \leftarrow \text{random}(0, D)$
    for $j \leftarrow 0$ to D **do** ▷ Generate a new mutated population
      if $\text{random}(0,1) < C_r$ **or** j is j_{rand} **then**
        $x, y, z \leftarrow \text{random}(0, N)$ **and** $x \neq y \neq z \neq i$
        newPars[$iD + j$] ← Pars[$xD + j$] + F_m(Pars[$yD + j$] − Pars[$zD + j$])
      else
        newPars[$iD + j$] ← Pars[$iD + j$]
      end if
    end for
    $J_{\text{new}}[i]$ ← COSTFUNCTION(newPars[i], Data_{qt}, Δt_{ql}, ΔX_{ql}) ▷ Evaluate the new population
    if $J_{\text{new}}[i] < J[i]$ **then** ▷ Selection of a better parameter set
      $J[i] \leftarrow J_{\text{new}}[i]$
      for $j \leftarrow 0$ to D **do** Pars[$iD + j$] ← newPars[$iD + j$]
      end for
    end if
  end parfor
end for

Finalization: Searches for the index i_{min} with the lower value in the vector J and returns the best estimate of the parameters in the population Pars[$i_{\text{min}}D + j$] where $j \leftarrow 0$ to D.
